# Number of long-term inpatients in Japanese psychiatric care beds: trend analysis from the patient survey and the 630 survey

**DOI:** 10.1186/s12888-020-02927-z

**Published:** 2020-11-03

**Authors:** Tatsushi Okayama, Kentaro Usuda, Emi Okazaki, Yoshio Yamanouchi

**Affiliations:** 1 Department of Neuropsychiatry, Osaka Medical College, 2-7 Daigakumachi, Takatsuki, Osaka, 569-8686 Japan; 2 Department of Mental Health Policy, National Institute of Mental Health, National Center of Neurology and Psychiatry, 4 Chome-1-1 Ogawahigashicho, Kodaira, Tokyo, 187-0031 Japan; 3 Aisei Century Hospital, 4-28 Soikecho, Minamiku, Nagoya, Aichi 457-8515 Japan

**Keywords:** Long-term inpatients, Psychiatric care beds, Future estimates, Fatalities, Community-based integrated care

## Abstract

**Background:**

The number of psychiatric care beds and the mean length of stay in psychiatric care beds in Japan have decreased over the past 10 years. However, as has long been indicated here and elsewhere, Japan lags behind other countries in terms of deinstitutionalization. Furthermore, the population of inpatients in psychiatric care beds is aging dramatically. In addition to the diversification of mental illness, the question of what measures to implement going forward regarding current psychiatric bed resources has emerged as a new challenge.

**Methods:**

Using data from the Patient Survey and the 630 Survey, we examined trends in the number of long-term inpatients in psychiatric care beds in Japan through 2040. Population estimation was used for estimating long-term hospital bed demand because of small fluctuations in the admission and discharge of long-term inpatients.

**Results:**

In 2017, nearly one-third of all long-term hospitalized patients were aged ≥75 years, and an estimated 47% of the total are expected to die by 2040. Thus, the overall demand for long-term hospitalization is forecast to decrease sharply due to aging of currently hospitalized long-term inpatients. The number of long-term inpatients in 2017 was 167,579, and this is projected to decrease to 103,141 in 2040.

**Conclusions:**

We believe it is necessary to adopt a multifaceted approach to promote hospital discharge and transition to the community, and to address the diversification of mental illness and the issue of psychiatric care bed supply/availability, which are forecast to decrease due to the natural decrease in long-term inpatients.

## Background

**Fig. 1:**
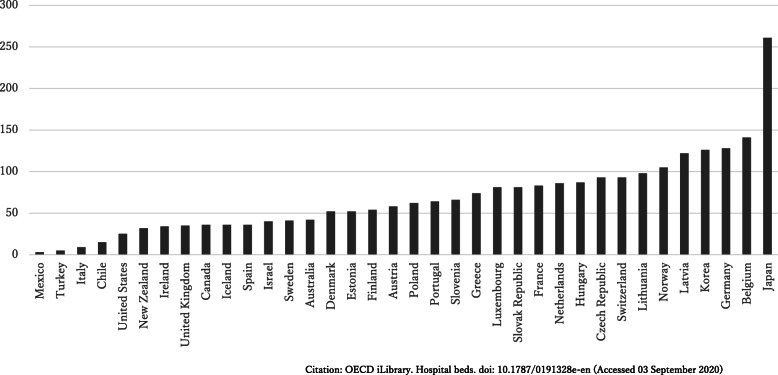
Psychiatric beds per 100,000 population. The number of psychiatric beds in Japan in comparison with those of other OECD countries

Japan has more psychiatric care beds than other Organisation for Economic Cooperation and Development (OECD) countries, a fact that has recently become a topic of discussion (Fig. 1) [1]. Japan has not gone through the process of reforming its system for allocating hospital beds, mainly because public funding is not available for privately funded hospitals. Consequently, long-term care beds that should have been categorized as beds in the community remain categorized as long-term care beds. This is one of the reasons Japan has more psychiatric care beds compared with other countries. As has long been indicated within Japan and elsewhere, Japan lags behind other countries in terms of deinstitutionalization [2–5]. The Japanese Ministry of Health, Labour and Welfare (MHLW) 2004 Vision for Reform of Mental Health and Welfare expressed the intent to reduce the number of long-term care beds [6]. Since then, the MHLW focused on transitioning to community mental health services with the stated goal of cutting back 70,000 psychiatric care beds in 10 years. However, from 2004 to 2018, the number of psychiatric care beds actually decreased from 356,000 to just 330,000, a reduction of approximately only 25,000 beds [7, 8].

The mean length of stay in psychiatric care beds in Japan (mean length of hospital stay among discharged patients) was approximately 500 days in 1990 but fell to under 300 days in 2011 and further decreased to approximately 266 days in 2018 [9]. This number is also conspicuously higher than in other OECD countries. However, as with the number of hospital beds, definitions differ by country. Still, thanks to an increased number of treatment facilities, advancements in drug therapy, and government policies based on development of infrastructure to promote transition to community mental health services, the average length of hospital stay has decreased by 50 days in the last 10 years, with a further decrease expected going forward [9]. In addition, hospital stay lengths among new admissions have recently decreased, with 86% of patients being discharged within 1 year [10].

As Japanese society has aged, the Japanese population has decreased from a peak of 128.06 million in 2010 to 126.44 million in 2018, with people aged ≥65 years accounting for 28% of the total population, the highest percentage ever [11]. The Japanese population will decrease more rapidly going forward and the population in 2040 is projected to be about 110.92 million people, 36% of whom will be aged ≥65 years [11]. Inpatients in psychiatric care beds are aging dramatically. An examination of the number of inpatients in psychiatric care beds by age group in a 2017 survey revealed that 58% of patients were aged ≥65 years [12]. In addition to the diversification of mental illness, the question of what measures to institute going forward about current psychiatric bed resources has emerged as a new problem.

With this current state of affairs, the question of future trends in long-term inpatients, who currently occupy more than half of psychiatric care beds, has become a major problem for health care policy and hospital management. In 2001, using statistics for psychiatric inpatients in Niigata Prefecture, Someya et al. conducted a time-series analysis of patients with schizophrenia and patients with other mental illnesses and published their projections of changes in the numbers of these patients over the next 20 years [13, 14]. This study focused on the fact that a large number of inpatients were born in a certain narrow timeframe. The inpatients were divided into age groups, and changes in the number of patients per age group were projected by applying the time-series characteristics of trends in each group [13]. Therefore, using data from the Patient Survey and the 630 Survey (which are described later), we examined trends in the number of long-term inpatients until 2040 using a projection method different from that employed by Someya et al. [13].

## Methods

**Fig. 2:**
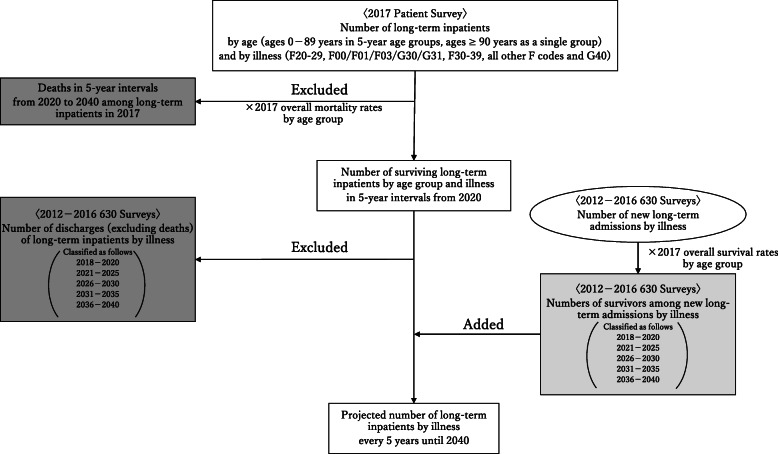
Projection method. We used the following formula: Projected number of long-term inpatients by illness = Total number of long-term inpatients in 2017 − Deaths among long-term inpatients in 2017^*1^ − Number of long-term discharges to determine discharge-admission differences.^*2^. ^*1^ Deaths among long-term inpatients in 2017 = Number of long-term inpatients by age group × General mortality rates by age group. ^*2^ Number of long-term discharges to determine discharge-admission differences = Number of discharges (excluding deaths) of long-term inpatients by illness − (Number of new long-term admissions by illness × 2017 overall survival rates by age group)

Future demand for hospitalization is typically projected by applying the rate of inpatient care in the overall population to the estimated future population [15]. However, this method is unsuitable for projecting long-term hospital bed demand because of the small fluctuations in the admission and discharge of long-term inpatients. Therefore, we adopted the cohort-component method, which is widely used in demography [16], to analyze trends in the number of long-term inpatients in psychiatric care beds in Japan. Long-term hospitalization was defined as a hospital stay lasting 1 year or more. Our projection method is shown in Fig. 2.

The total number of long-term inpatients in 2017 and the number of long-term inpatients by age group were taken from the 2017 Patient Survey [17]. The Patient Survey is an official statistical survey conducted once every 3 years by the MHLW that randomly samples patients from medical facilities throughout Japan to create a snapshot of the injuries and illnesses of these patients. The survey is conducted on a certain date in mid-October that differs for each facility. According to the 2014 Patient Survey, the sampling rate was approximately 38%. In this study, we independently aggregated data from the Patient Survey that was provided to us by the MHLW in accordance with Article 33 of the Japanese Statistics Act of 2017 because we wanted to incorporate data beyond that which is publicly available.

We used the 630 Survey to determine the number of long-term discharges and new long-term admissions to be used for calculations of discharge-admission differences [12]. The 630 Survey is a snapshot of patients using psychiatric hospitals, psychiatric clinics, and home-visit nursing services on June 30 (i.e., 6/30) every year. It is a statistical survey conducted to obtain information to promote mental health and welfare policies. The response rate exceeds 95%, and thus the 630 Survey is highly exhaustive.

For both the Patient Survey and the 630 Survey, we divided mental illnesses into four categories: schizophrenia, dementia, mood disorder, and “other”. These mental illnesses were defined as the following International Statistical Classification of Disease and Related Health Problems (10th revision) codes: F20–29 for schizophrenia; F00, F01, F03, G30, and G31 for dementia; F30–39 for mood disorder; and all other F codes and G40 for “other.” Regarding mood disorders, we could not segment unipolar depression and bipolar disorder because they were not separately classified in the 630 Survey. For age groups in the Patient Survey, ages 0–89 years were divided into 5-year age groups, while all ages ≥90 years were treated as a single group. For age groups in the 630 Survey, we used the classifications in the original survey, although they are approximations: < 20 years, 20–39 years, 40–64 years, 65–74 years, and ≥ 75 years.

General mortality rates by age group were determined with the 2017 Report on Vital Statistics. Reports on Vital Statistics are published annually by the MHLW to determine the demographics of the Japanese population, including foreigners. General survival rates by age group were determined by subtracting the general mortality rates by age group from 1.

Calculations were made in three stages. First, we estimated deaths by age group based on long-term inpatient numbers in the 2017 Patient Survey and general mortality rates by age group listed in the 2017 Report on Vital Statistics. Long-term inpatient numbers were classified by illness and age group. For general mortality rates by age group, based on the assumption that current trends will continue, we used the same numbers to project from 2017 onwards. Associated statistics were multiplied by the previously described general mortality rates by age group to estimate future deaths for each illness and age group. Deaths of patients who are long-term inpatients as of 2017 were calculated in five-year intervals. These numbers were then added together, and the results were divided into those for ages ≤74 years and ≥ 75 years.

Next, for discharge-admission differences, we subtracted the “number of new long-term admissions” from the “number of long-term inpatients discharged for reasons other than death” in the 2012–2016 630 Survey; future differences were estimated as the average difference for the past 5 years. Thus, the 2017 difference was estimated as the average difference for 2012–2016, the 2018–2020 difference was estimated as the average difference for 2013–2017, and the 2021–2025 difference was estimated as the average difference for 2016–2020. For 2026–2030, 2031–2035, and 2036–2040, we used the average difference for 2021–2025. Considering that deaths will also occur among new long-term admissions, we classified new long-term admission numbers by age group and multiplied them by general survival rates by age group (determined as described earlier) to determine the number of survivors among new long-term admissions. We then subtracted the number of survivors among new long-term admissions from the number of long-term discharges to determine discharge-admission differences.

Lastly, we subtracted the number of deaths and discharge-admission differences (calculated as described above) from long-term inpatients (as of 2017) to estimate the number of demands for long-term hospitalization by illness.

## Results

**Fig. 3:**
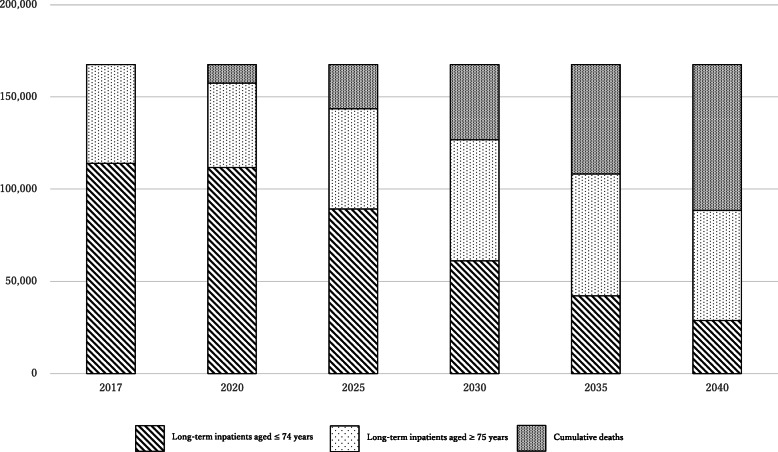
Projected number of fatalities. In 2017, there were a total of 167,579 long-term inpatients, nearly half of which (79,016) were projected to die by 2040

**Fig. 4:**
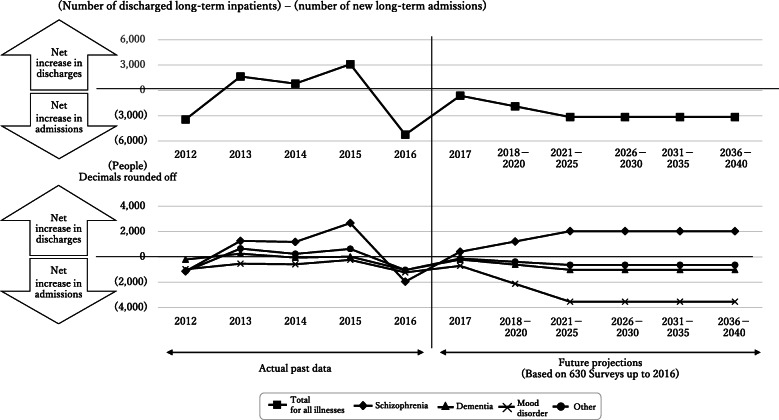
Projected number of discharge–admission differences. The number of admissions for all illnesses other than schizophrenia is projected to increase from 2017 onwards. The number of admissions is projected to exceed the number of discharges in terms of total number of illnesses from 2017 onwards

The results are shown in Figs. 3 and 4 and in Table 1.

**Table 1:** Projections for long-term hospitalization demand

Year	2017	2020	2025	2030	2035	2040
Disorder						
Schizophrenia	110,439	105,148	96,985	86,538	73,665	58,741
Mood disorder	9727	11,164	13,730	16,075	18,297	20,535
Dementia	29,505	25,723	21,011	16,395	12,726	10,342
Other	17,908	17,480	16,943	16,079	14,913	13,524
Total	167,579	159,515	148,668	135,087	119,602	103,141

(number of persons)

Figure 2 shows the number of long-term inpatients for the previous year minus the number of deaths. In 2017, there were a total of 167,579 long-term inpatients, 114,089 (68%) of whom were aged ≤74 years and 53,490 (32%) of whom were aged ≥75 years. However, of all long-term inpatients, nearly half (79,016) were projected to die by 2040. The number of long-term inpatients aged ≥75 years is projected to rise and fall repeatedly from 2017 onwards, peaking at 66,028 in 2035 and then decreasing to 59,750 in 2040. As the majority of long-term inpatients aged ≤74 years will grow older, this will result in a major decrease in the number of people in this age category. In 2040, the number of long-term inpatients aged ≤74 years is projected to be 28,812, approximately one-fourth of the 2017 number.

Figure 3 shows discharge-admission differences. Future differences were projected as the averages for the previous 5 years as described earlier and take into account deaths among new long-term admissions as well. In terms of overall numbers, in 2012, the number of admissions exceeded the number of discharges. From 2013 to 2015, the number of discharges exceeded the number of admissions. However, in 2016, the number of admissions for all illnesses exceeded the number of discharges. Due in part to this 2016 fluctuation, the projected number of admissions for all illnesses other than schizophrenia increased from 2017 onwards, with a dramatic increase in the number of patients admitted for mood disorder. The number of patients admitted for mood disorder had also increased prior to 2016, suggesting that long-term hospitalization of patients with mood disorder may present a problem going forward. In terms of the total numbers for all illnesses, the number of admissions is projected to exceed the number of discharges from 2017 onwards.

Table 1 shows projections for long-term hospitalization demand based on a combination of Figs. 2 and 3. In terms of numbers by illness, 9727 patients were hospitalized for mood disorder in 2017. This number was projected to increase annually to 20,535 in 2040, more than double the number in 2017. However, the number of long-term inpatients for all other illnesses was projected to decrease. While the number of long-term inpatients with dementia was 29,505 in 2017, this number was projected to decrease to 10,342 in 2040, approximately one-third of the 2017 number. The number of long-term inpatients in the “other” category was 17,908 in 2017 but was projected to gradually decrease thereafter to 13,524 in 2040. The number of long-term inpatients with schizophrenia was projected to decrease dramatically from 110,439 in 2017 to 58,741 in 2040, about half of the 2017 number. The decrease from 2030 to 2040 was especially notable. Overall, while admissions were projected to increase based on the results for discharge-admission differences, the demand for long-term hospitalization was forecast to decrease sharply due to the aging of long-term inpatients who are currently hospitalized. While the number of long-term inpatients in 2017 was 167,579, this number was projected to decrease to 135,087 (about 80%) in 2030 and to 103,141 (about 60%) in 2040.

## Discussion

In 2001, focusing on the fact that the number of hospitalized patients born between 1945 and 1949 is consistently high, Someya et al. [14] predicted that based on the aging of psychiatric inpatients associated with aging of the above-mentioned population and the major decline in the number of patients in that generation 30 years later, the number of inpatients with schizophrenia would decrease by at least 60% by 2030. As of 2018, the number of inpatients with schizophrenia has declined exactly as predicted [18] Also, a look at the overall number of psychiatric inpatients shows that the number in the 2017 Patient Survey is nearly the same as that calculated by Someya et al. [14, 17].

Why, then, was the number of hospital patients born between 1945 and 1949 always so high around that time? We believe that the answer may be related to the Seishinka Tokurei, a special measure related to hospital staffing issued in 1958 by the then Ministry of Health and Welfare. This measure permitted psychiatric wards to have only one-third as many doctors and two-thirds as many nurses as general hospital wards. Although the medical service fees were low, the ability to keep labor costs low meant that having long-term inpatients was a shortcut to stable hospital management. This state of affairs led to a vast increase in psychiatric care beds in private hospitals, which resulted in a large number of psychiatric patient admissions from the 1960s to the 1980s. The group of inpatients among these admissions whose hospital stays became long-term are considered to account for the majority of long-term inpatients today. Another special law related to psychiatric medicine, the Act on Mental Health and Welfare for the Mentally Disabled, is in place today. The psychiatric health care system operates under this system, thereby allowing psychiatric hospitals to be managed at lower costs than general hospital beds [19].

However, long-term hospitalization causes health care costs to balloon and is thus undesirable from the perspective of promoting transition to community mental health services, which emphasize quality of life. Therefore, as part of the Vision for Reform of Mental Health and Welfare, the MHLW has long strived to ensure transition to community mental health services with the stated goal of cutting back 70,000 psychiatric care beds in 10 years. Despite this goal, only approximately 25,000 beds have been eliminated in 15 years. Although Japan has been transitioning to community mental health services as other countries have, this transition has not yet led to a major reduction in long-term psychiatric care beds. According to the WHO Mental Health ATLAS 2017, the percentage of newly admitted patients whose stays become long-term (1 year or more) has decreased to roughly 12% [20]. However, more than two-thirds of all patients in psychiatric care beds overall stay for more than 1 year [12]. Factors behind the continuation of long-term hospitalizations likely include cultural and societal factors such as stigma.), a lack of use of clozapine, and a lack of resources such as outreach and home-visit nursing services [21]. Another conceivable factor is the absence of financial compensation for private hospitals. Clozapine, which was introduced in Japan in 2009, has made almost no penetration since then; in January 2018, clozapine was prescribed for only 0.1% of all patients with schizophrenia [12]. Thus, clozapine has unfortunately not led to reduced rates of treatment-resistant schizophrenia or long-term use of psychiatric care beds. Also, due to sharp increases in the incidence of dementia and mood disorders such as depression, the number of patients with mental illness has increased by 60% in the last 15 years, breaking the 4-million mark and reaching 4.193 million in 2017 [17]. Going forward, hospital stays must be further shortened, and intervention must begin earlier. Shortening hospital stays would greatly reduce the number of psychiatric care beds.

While social factors such as the increasing application of information technology are also conceivable reasons for the increase in patients with mood disorders, other factors are likely greater; specifically, a change in the concept of depression and other mood disorders, and higher frequencies of consultation due to the spread of this change. In addition, the reason that 20,000 patients with mood disorders are predicted to require long-term psychiatric care beds going forward is not because those patients have mood disorders but because 12% of patients with all diseases in Japan are chronic patients [20]. Therefore, as the overall number of patients increases, the number of patients who require long-term psychiatric care beds is also predicted to increase.

The reduction in dementia-related psychiatric care beds is thought to be due largely to the transfer of patients with dementia to aged care facilities. However, due to the absence of connections among medical, welfare, and care data, a clear conclusion cannot be drawn. Therefore, surveys investigating these issues need to be conducted in the future. However, in Japan, there is a great deal of movement back and forth between aged care facilities and psychiatric hospitals due to factors such as persons living in aged care facilities being placed in psychiatric hospitals as soon as they demonstrate psychiatric symptoms or behavioral and psychological symptoms of dementia. This high degree of movement is a factor in the predicted reduction of dementia-related long-term psychiatric care beds. The stigma attached to psychiatric patients may also play a role.

The predicted closure of 64,000 long-stay beds is likely to have substantial financial implications. Specifically, with the lack of public funding for private hospitals and a dwindling population, hospitals will be forced to downsize and focus more on outpatient care to remain operational. However, due to the lack of experience with community mental health services in Japan, preparing such services would likely take time. The Japanese government, in particular the MHLW, is currently advancing a policy of comprehensive regional care systems that can also handle psychiatric disorders. This policy aims to promote not only the development of infrastructure to facilitate transition to community mental health services but also to promote therapeutic drugs for treating treatment-resistant schizophrenia and measures for managing dementia to realize discharge and community transition. However, this policy only began in 2017 and has not yet produced structural change. In light of this present situation, we believe it is necessary to leave behind the mindset that “long-term hospitalization is bad” and adopt a multifaceted approach to the diversification of mental illness and the issue of psychiatric bed supply/availability, which are forecast to decrease going forward due to the natural decrease in long-term inpatients.

Nevertheless, the Patient Survey used in this study is conducted only once every 3 years and is not very exhaustive, with a sampling rate of only about 38%. Thus, the survey does not encompass all long-term psychiatric inpatients. Another point that must be noted regarding the Patient Survey is that it represents the number of patients at a specific point in time, not for an entire year. Furthermore, our discharge-admissions differences represent the mean differences of 5-year intervals based on data from 630 Surveys up to 2016. Consequently, it must be said that differences for 2017 onwards are uncertain. In addition, there is no publicly available data for future forecasts of mortality rates. Therefore, models are forced to incorporate the assumption that current mortality rates will remain unchanged. While it is assumed that current mortality rates will remain unchanged until 2040, advancements in medicine could extend lifespans, making it highly likely that the number of patients will increase by 2040.

We are currently striving to integrate care to create a health care system centered on community-based integrated care [22]. Several other countries, particularly in Western Europe, have already been actively transitioning from institutional care to community-based care [23, 24]. Based on our findings in this study and on these efforts in other countries, we conclude that it may be pertinent to consider the comprehensive structure of future mental health care.

## Conclusions

Reducing the number of psychiatric inpatients and the length of hospital stays has long been a topic of discussion in Japan. In this study, we used the Patient Survey and the highly exhaustive 630 Survey to project the future demand for long-term hospitalization. We predict that a sharp rise in deaths due to population aging will reduce the demand for long-term hospitalization in 2040 to approximately 60% of that recorded in 2017. The results of this study raise the question of what actions to take in order to address the effects that this reduction in hospital care beds, which will occur more as a result of natural forces than of government policy.

## Data Availability

The datasets analyzed during the current study are available at the following website: https://www.ncnp.go.jp/nimh/seisaku/data/630/. The Patient Survey data used in this study was provided to us by the MHLW in accordance with Statistics Act of 2007, Article 33; therefore, we are unable to publish these data ourselves and the MHLW has not made the data publicly available online. The 630 Survey is publicly available data that is accessible and available to anyone.

## Abbreviations

OECD: Organisation for Economic Cooperation and Development
MHLW: Ministry of Health, Labour and Welfare

## References

[1] Allison S, Bastiampillai T, Licinio J, Fuller DA, Bidargaddi N, Sharfstein SS (2018). When should governments increase the supply of psychiatric beds?. Mol Psychiatry.

[2] Hospital Authority (2011). Mental health service plan for adults 2010–2015.

[3] Ito H, Sederer LI (1999). Mental health services reform in Japan. Harv Rev Psychiatry.

[4] Nicaise P, Dubois V, Lorant V (2014). Mental health care delivery system reform in Belgium: the challenge of achieving deinstitutionalization whilst addressing fragmentation of care at the same time. Health Policy.

[5] World Health Organization (2001). The World Health Report 2001 - Mental health: New understanding, new hope.

[6] Japan Ministry of Health, Labour and Welfare (2004). Vision for reform of mental health and welfare.

[7] Japan Ministry of Health, Labour and Welfare (2018). Medical facility survey.

[8] Tachimori H, Takeshima T, Kono T, Akazawa M, Zhao X (2015). Statistical aspects of psychiatric inpatient care in Japan: based on a comprehensive nationwide survey of psychiatric hospitals conducted from 1996 to 2012. Psychiatry Clin Neurosci.

[9] Japan Ministry of Health, Labour and Welfare (2018). Hospital report.

[10] Okumura Y, Sugiyama N, Noda T, Tachimori H (2019). Psychiatric admissions and length of stay during fiscal years 2014 and 2015 in Japan: a retrospective cohort study using a nationwide claims database. J Epidemiol.

[11] National Institute of Social Security and Population Research (2017). Projection of Japanese population [in Japanese].

[12] National Institute of Mental Health, National Center of Neurology and Psychiatry, Japan (2017). Annual data of mental health in Japan.

[13] Someya T, Suzuki Y, Pak C, Sham PC, Tang SW (2004). Forecasting the number of inpatients with schizophrenia. Psychiatry Clin Neurosci.

[14] Suzuki Y, Someya T (2001). Annual transition of the number of inpatients and projection in coming twenty years [in Japanese]. J Japan Assoc Psychiatric Hosp.

[15] Doi S, Ide H, Takeuchi K, Fujita S, Takabayashi K (2017). Estimation and evaluation of future demand and supply of healthcare services based on a patient access area model. Int J Environ Res Public Health.

[16] Smith S, Tayman J, Swanson D (2013). Overview of the cohort-component method. State and Local Population Projections. The Springer Series on Demographic Methods and Population Analysis.

[17] Japan Ministry of Health, Labour and Welfare (2017). Patient Survey.

[18] Someya T. Formulating mental health care policy based on scientific evidence: future trends based on the evolution of methods for estimating hospital bed numbers, predictions of future numbers of patients with schizophrenia, and the basis for and validity of the development of estimation methods. Psychiatria et Neurologia Japonica. 2019;311. (article in Japanese).

[19] Kasai K, Ando S, Kanehara A, Kumakura Y, Kondo S, Fukuda M (2017). Strengthening community mental health services in Japan. Lancet Psychiatry.

[20] World Health Organization (2018). Mental Health ATLAS 2017 Member State Profile.

[21] Oshima I, Mino Y, Inomata Y (2007). How many long-stay schizophrenia patients can be discharged in Japan?. Psychiatry Clin Neurosci.

[22] Naruki H (2016). Examining the importance of and identifying methods to promote “integration” for structuring community-based integrated care systems. J Natl Inst Public Health.

[23] Alwan NA, Johnstone P, Zolese G (2008). Length of hospitalisation for people with severe mental illness. Cochrane Database Syst Rev.

[24] Ravelli DP (2006). Deinstitutionalisation of mental health care in the Netherlands: towards an integrative approach. Int J Intgr Care.

